# On-Chip Multiple Particle Velocity and Size Measurement Using Single-Shot Two-Wavelength Differential Image Analysis

**DOI:** 10.3390/mi11111011

**Published:** 2020-11-17

**Authors:** Shuya Sawa, Mitsuru Sentoku, Kenji Yasuda

**Affiliations:** 1 Department of Physics, School of Advanced Science and Engineering, Waseda University, Tokyo 169-8555, Japan; tkdc10@moegi.waseda.jp (S.S.); mitsen1019@fuji.waseda.jp (M.S.); 2 Department of Pure and Applied Physics, Graduate School of Advanced Science and Engineering, Waseda University, Tokyo 169-8555, Japan

**Keywords:** imaging flow cytometer, precise velocity measurement, single-shot image-based velocity measurement, particle shape reconstruction, multi-view imaging, exposure time difference

## Abstract

Precise and quick measurement of samples’ flow velocities is essential for cell sorting timing control and reconstruction of acquired image-analyzed data. We developed a simple technique for the single-shot measurement of flow velocities of particles simultaneously in a microfluidic pathway. The speed was calculated from the difference in the particles’ elongation in an acquired image that appeared when two wavelengths of light with different irradiation times were applied. We ran microparticles through an imaging flow cytometer and irradiated two wavelengths of light with different irradiation times simultaneously to those particles. The mixture of the two wavelength transmitted lights was divided into two wavelengths, and the images of the same microparticles for each wavelength were acquired in a single shot. We estimated the velocity from the difference of its elongation divided by the difference of irradiation time by comparing these two images. The distribution of polystyrene beads’ velocity was parabolic and highest at the center of the flow channel, consistent with the expected velocity distribution of the laminar flow. Applying the calculated velocity, we also restored the accurate shapes and cross-sectional areas of particles in the images, indicating this simple method for improving of imaging flow cytometry and cell sorter for diagnostic screening of circulating tumor cells.

## 1. Introduction

Numerous microfluidic devices have been used for cellular analysis technologies in biological research and medical diagnoses [1,2,3,4,5,6,7,8]. Recently, the change of cells’ morphological characteristics as they pass through the cell cycle was used to sort particular yeasts in a fully automated low-cost imaging cell sorter with a cell detection rate of 5 s [9]. A low-cost microfluidic eight-way simultaneous image data-based sorting system mounted on a fluorescence microscope was also demonstrated with a sorting speed of 0.5 s resolution [10]. Furthermore, an automated imaging cell sorter system for cell identification within a flowing droplet was recently reported [11]. For the improvement of image analysis abilities, a deep-learning-assisted imaging cell sorter system was documented [12], in which the abilities of image analysis and cell identification were significantly improved while maintaining a time resolution on the sub-second order. Another unique machine learning-based approach for identifying of target cells with direct diffraction detection and analysis without any cell image acquisition was proposed in [13].

Image and size information of cells were applied for detection and identification of target cells for diagnostic applications [3,14,15,16,17], and recently, three-dimensional (3-D) images of single cells and their clusters were acquired [18]. However, the challenges of establishing a precise high-throughput for those technologies remained an issue and must be improved. Hence, the precise high-throughput image cell sorter combining with those existing methods can give us more flexible new approaches to medical diagnosis. For example, in our previous studies, we proposed an original approach for circulating tumor cell (CTC) detection in blood based on analyses of the morphometric parameters of cells without any antibody staining, with these parameters acting as “imaging biomarkers” [19,20,21,22]. For this approach, we developed a high-speed cell image recognition and analysis system to identify the target cells using their morphological characteristics during their flow within a narrow microfluidic pathway for sorting. We also improved this system and developed an on-chip multi-imaging flow cytometry system [23,24,25], which allows the simultaneous acquisition of both bright-field (BF) and fluorescent (FL) images when scanning all cells in the blood. Using this system, clustered CTCs were accurately detected for identifying their cluster sizes and their nucleus shapes and numbers within clusters simultaneously in the blood of cancer cell-implanted rats.

In contrast to the significant advancement in image recognition and analysis technologies in the field of imaging cell sorting, the fundamental and critical technical issues of the cell sorting process, such as the precise measurement of microparticle flow speed for correct target collection, has had no significant progress. For example, in our previous studies, only the mean values of the estimated flow velocities were applied for sorting timing set-up. Moreover, the influence of the exposure time of image acquisition, which is the origin of image blur, was neglected for the precise measurement of the acquired shapes of targets or their size estimations; we desired a method to overcome this limitation for more accurate and strict measurement and sorting of target samples.

We report the ability and limitation of our newly developed particle flow velocity measurement method with two-wavelength simultaneous one-shot differential image analysis. The results showed that this method could acquire the velocities of all of the spread particles in the microfluidic flow simultaneously only with a one-shot of image acquisition. In contrast, the conventional measurement was based on the two-shot image comparison for single target samples. The acquired particle velocities were also applied for the correction of the cross-sectional areas of particles, which were elongated by their movement during the exposure time of image acquisition. The ability and limitation of this method caused by shutter-time and image pixel size resolution of hardware for this analysis method were also discussed.

## 2. Principle

First, we explain the principle of the single-shot simultaneous two-wavelength differential imaging analysis for precise particle flow velocity measurement. In this method, we exploited the difference of exposure time of two light sources for image acquisition effectively for calculating the flow velocities of particles within the microfluidic pathway by measuring the difference of elongation length of the two images of the same particle by only a single shot of image acquisition interval. Usually, the exposure time is necessary to acquire the image and is also the reason for image blur, which is caused by the movement of targets within the exposure time. However, in this method, we used this image blur effectively to determine the precise flow velocities of particles. When we irradiate two different wavelength of lights with different lengths of time to the flowing particle, the acquired length of the particle should be elongated to the direction of flow depending on the exposure time of image acquisition. Hence, we can compare the length of the particle in two images of different wavelengths and can estimate the velocity by the difference of irradiation time of two-wavelength lights. Therefore, in this method, we only need one shot of image acquisition for velocity analysis and can apply this method to all the particles within a picture simultaneously without any complicated procedure of analysis. The advantages of this method are (1) only a single shot of image acquisition interval is needed for the estimation of flow velocity; (2) the velocities of all the particles within an image can be analyzed simultaneously; (3) the ability to reconstruct the actual image data from the accurate flow velocities to overcome the influence of image blur; (4) the system is easily set-up (only two different wavelengths, the exposure time of light source, and image dividing unit are needed).

**Figure 1:**
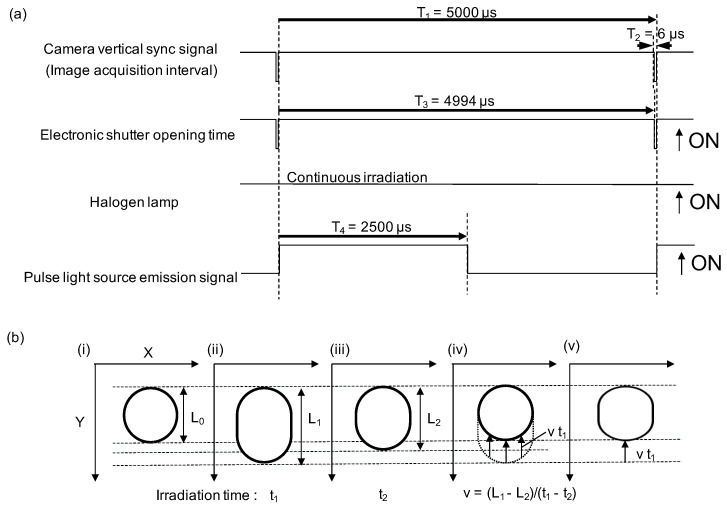
Restoration principle of accurate particle shape. (**a**) The time scale of the camera’s image acquisition interval, electronic shutter, externally inserted halogen lamp, and a light emitting diode (LED) pulsed light signal. (**b**) Particle images acquired by photographing with different irradiation times with two restoration principles. (i) Image of a stationary particle. (ii), (iii) Images of particles moving in the *Y*-axis direction taken at irradiation time $t_{1}$ and $t_{2}$ ($t_{1}>t_{2}$), respectively. The different irradiation times cause the difference in lengths ($L_{1}$, $L_{2}$). The velocity of moving particles can be calculated from the difference in irradiation time and lengths. (iv) Image restoration processing of the accurate particle shape. The lower part of the elongated particle is lifted by the amount of elongation determined by multiplying the calculated velocity and irradiation time. (v) The shape of the particle is restored by the conventional restoration principle that presses the Y-direction for the amount of elongation.

Figure 1 is a schematic drawing explaining the principle of the method applied for our experiments as an example. Figure 1a illustrates the time chart of the camera’s image acquisition interval, electronic shutter, externally inserted halogen lamp with a band-pass filter (590 nm), and a light emitting diode pulse (LED) signal source (530 nm). The frame rate of the Charge-Coupled Device (CCD) camera is 200 frames per second (fps), so one image is acquired every 5000 μs. The camera’s electronic shutter, adjustable between 2 and 4994 μs, limits the exposure time of the halogen lamp that continues to irradiate. As a second light source, an LED with a period of 5000 μs (irradiation time: 2500 μs) synchronized with the image acquisition timing is irradiated.

Figure 1b illustrates the principle of this method to acquire the actual images of moving target samples: First, the image of the round shape sample (i) is elongated by the flow (movement) during an interval of image capture frame (ii), (iii). Simultaneous irradiation of two different wavelengths at irradiation times $t_{1}$ and $t_{2}$ ($t_{1}>t_{2}$) causes a difference in lengths ($L_{1}$, $L_{2}$), respectively. The particle’s velocity can be calculated from the difference in lengths and irradiation times. The elongation of the particle image is determined by multiplying the calculated speed and irradiation time. The accurate shaped particles’ image is acquired by image processing, where the lower part of the elongated particle is lifted by the amount of the elongation (iv). On the other hand, it is impossible to restore the correct shape with the conventional image processing method that solely considers the vertical scale and compresses the flow direction (*Y*-axis) equally (v).

## 3. Materials and Methods

### 3.1. Measurement System

The measurement system consists of seven main parts: two light sources, a dichroic mirror unit for light mixing, a microfluidic chip (microchip), an objective lens, a multi-view module to divide an image into the two images of different wavelengths, a CCD camera camera, and a computer. A halogen lamp (LG-PS2; Olympus Co., Tokyo, Japan) was used for the 590 nm light source with 590 nm band-pass filter. An LED light (LED4D069, THORLABS, Newton, NJ, USA) was used for another 530 nm light source. The magnitude of the objective lens (×20, UPLSAPO, Olympus Co., Tokyo, Japan) was chosen to meet the microchannel width of the microchip. The multi-view module was custom made having a two-wavelength image dividing function. A high-speed digital CCD camera (HXC13; Baumer, Friedberg, Germany) was used for every 5000 μs image acquisition.

### 3.2. Microfluidic Chip

A disposable microfluidic chip (microchip) was fabricated with polydimethylsiloxane (PDMS) (SYLGARD 184 silicon elastomer; Dow Corning Co., Midland, MI, USA) attached to a thin glass slide by the same procedure as we reported previously [23], and was used for monitoring. In the microchip, the upper stream was divided into three channels: the central one was used for the sample inlet, and the remaining two side channels were used for sheath buffer inlets. After the junction at which the sample and sheath flow meet, the width of the sample flow was focused by hydrodynamic focusing, which allowed imaging of every single particle upon the arrangement of all of the particles in a straight line. The hydrodynamic focusing in this chip design conferred several advantages, such as centering the samples in the microfluidic flow, adding spaces between neighboring samples when lining them up, and aligning the orientation of samples in the direction of flow.

### 3.3. Preparation of Samples

In this experiment, two types of samples were used. Micro polyethylene spheres (diameter was 12 μm, Thermo Fisher Scientific K.K., Tokyo, Japan) were used as the standard size microparticle. They were diluted with pure water (6.38 $\times 10^{5}$ beads/mL) and injected into the sample inlet of the microfluidic chip. Human cervix epithelioid carcinoma HeLa cells (ATCC, Manassas, VA, USA) were used as the standard cell sample. They were cultured in Dulbecco’s Modified Eagle Medium (DMEM, Thermo Fisher Scientific K.K., Tokyo, Japan) with 10% Fetal Bovine Serum (FBS, Thermo Fisher Scientific K.K., Tokyo, Japan) and 1% Penicillin-Streptomycin (Thermo Fisher Scientific K.K., Tokyo, Japan). They were adjusted with culture medium (6.00 $\times 10^{4}$ cells/mL) and injected into the sample inlet.

### 3.4. Lithography Processing with SU-8

Micropatterns designed by CAD were drawn on the mask blanks with a laser lithography system. Then, a photoresist layer and chromium layer were removed. After the SU-8 (SU-8 3000, KAYAKU Co. Ltd., Tokyo, Japan ) coating process, the substrate was soft-baked to evaporate the solvent. Soft baking temperature and duration were $95\;{}^{\circ }C$ and 5 min, respectively. The substrate with a photomask was then exposed to irradiation under a UV lamp to induce cross-linkage. After the irradiation process, a Post-Exposure Bake (PEB) was performed. Post exposure baking had two steps. At the first step, temperature and duration were $65\;{}^{\circ }C$ and 1 min. At the second step, temperature and time were $95\;{}^{\circ }C$ and 5 min. Ultimately, the SU-8 resist that remained unhardened was removed by the SU-8 developer.

### 3.5. Lithography Processing with PDMS

Liquid PDMS was placed in the middle of the SU-8 mold and applied pressure for 15 min to uniformly spread the PDMS from the center to the whole SU-8 pattern. To harden the applied PDMS, the SU-8 mold was baked at $65\;{}^{\circ }C$ for 1 h. After the PDMS substrate became hardened, the PDMS was peeled from the mold carefully.

### 3.6. Program Construction of Velocity Measurement and Image Processing for Accurate Shape Restoration

Several steps were taken to obtain the length in the *Y*-axis direction of the particles. Firstly, the background images from the two images of the particle (captured by two different wavelength lights) were subtracted. Thereafter, these images are binarized by a threshold set in consideration of the gray-scale of the particles. As these binary images are likely to contain noise cracks, the morphological operation must be conducted to eliminate these imperfections. We counted the most extended *Y*-axis length of each particle and calculated the velocity based on the principle.

Next, the correct shapes of the particles were restored by utilizing the calculated velocity. By multiplying the speed and the light irradiation time, the extensions of the particles can be calculated. As the amount of the elongations are determined by this procedure, copying the gray-scale values of the parts that were subjected to elongation back to the original positions will restore the original shape and area of the flowing particles. The area was determined from the binary image, and a comparison was made before and after the processing. All of this was done by Python 3.8 (Python Software Foundation (PSF), Wilmington, DW, USA).

## 4. Results and Discussion

### 4.1. Velocity Evaluation with Simultaneous Acquisition of the Two Images with Different Exposure Time

**Figure 2:**
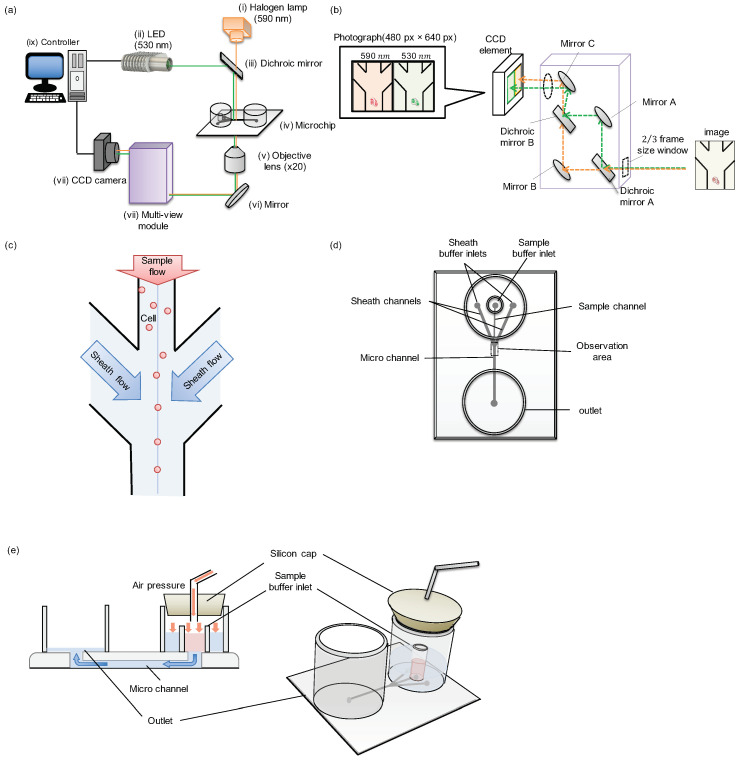
Set-up of the image acquisition flow cytometer with simultaneous two-wavelength differential imaging. (**a**) Equipment diagram of image acquisition flow cytometer system using simultaneous two-wavelength differential imaging. The system consists of nine modules. (i) Halogen lamp with band-pass filter (wavelength: 590 nm, irradiation time: 4994 μs); (ii) LED (wavelength: 530 nm, irradiation time: 2500 μs); (iii) Dichroic mirrors that reflect light below 550 nm—it was used for inserting LED in the light path of the halogen lamp; (iv) microchip; (v) objective lens (×20); (vi) mirror; (vii) multi-view unit; (viii) high-speed Charge-Coupled Device (CCD) camera (200 fps); (ix) controller. (**b**) Multi-view unit schematic diagram. The device adopts the mirror and the dichroic mirrors to enable the separation of an image into two at a wavelength of 550 nm. (**c**) An image of cells flowing through a flow cytometer. The cells converge in the flow channel with the sheath buffer. (**d**) Microchip schematic diagram. The microchip consists of polydimethylsiloxane (PDMS) on which the tubes are attached to the surface to create the inlets and the outlets. (**e**) The schematic diagram of the sample flows into the microchannel using air pressure.

To acquire two pictures of different wavelengths simultaneously, we fabricated the following set-up of the system. Figure 2a is a schematic illustration of the image acquisition flow cytometer with simultaneous two-wavelength differential image acquisition and analysis. The image acquisition interval was 5000 μs, and the two light sources were irradiated in synchronization. The irradiation time of the halogen lamp was set to 4994 μs by the camera’s electronic shutter, and the LED was set to 2500 μs. These two lights were mixed at the dichroic mirror unit and followed the same optical path, and irradiated the sample in the microchip. The image of particles within the microchannel of the microchip was acquired by the 20× objective lens. After passing through the multi-view unit, the mixed image was separated into two pictures of different wavelengths of light and aligned to the CCD camera side-by-side after cutting each image to meet the 1/2 size of the CCD module area. By the above process, an image with two wavelengths of light can be acquired simultaneously in a single shot. The difference of their elongation can be compared side-by-side only within a picture of CCD image recording.

Figure 2b shows the optical path image diagram inside the multi-view unit. The light that was a mixture of two-wavelength was passed through the 2/3 frame window for cutting the acquired image to meet the size of 1/2 of the CCD module area, and was separated into two different wavelengths by the dichroic mirror A. These lights were controlled by the adjusted mirrors and were positioned to the left half or right half of the CCD camera’s detector to be aligned side by side.

Figure 2c is an image of cells flowing through a microchannel in a microchip. In this experiment, we adopted hydrodynamic focusing to compare the distribution of particles before and after focusing. The samples were applied to the upper center stream and focused at the center of the pathway by the two side sheath flows. Then the samples were gathered to the center in the downstream area.

A schematic diagram of the microchip is described in Figure 2d. The sample and sheath buffer was placed in specified inlets, and the same air pressure was applied to these three inlets simultaneously to flow them into the microchannel equally.

Figure 2e shows the cross-sectional view of sample flow in the microchannel using air pressure. After the sample and the sheath buffers were inserted inside the specific inlets, a silicon cap lid was attached to seal the whole three inlets of the channels to apply the same air pressures for the balance of flow speeds. The samples and the sheath buffers were pushed by the applied air pressure and flow into the microchannels. The applied air pressure was controlled by the syringe pump and monitored. For example, when the applied pressure was 1.5 kPa, the max flow velocity at the center of the pathway was around 1 mm/s.

First, we examined the principle of velocity measurement with fixed 12 μm polystyrene spheres under the dry condition on the mechanically controlled movement apparatus. A beam chopper (MC1000; THORLABS, Newton, NJ, USA) was inserted to the position of the microchip within the system and the movement of the particle fixed in the microchip on the beam chopper was observed.

For the velocity measurement, the images of samples were cut around the particle at 80 pixels (px) × 80 px from the full size of images of 530 and 590 nm wavelengths, and those images of the particles at two wavelengths with different irradiation times were saved as PNG files into the analysis computer. After the subtraction of the background with no prerecorded sample images, the 8-bit (256-step) grayscale values of obtained particle images were transformed into binary images using threshold values based on the average intensities of the acquired images of each wavelength. Using binarized images, the lengths of the *X*-axis, *Y*-axis, and the areas of particles were determined by counting the white pixels.

**Figure 3:**
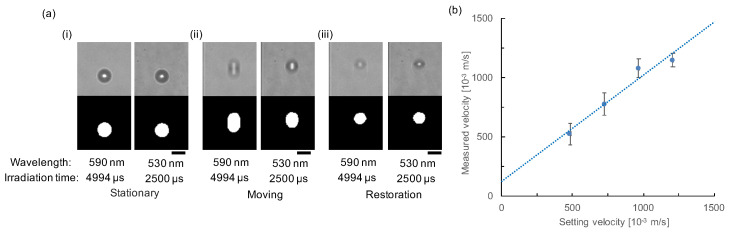
Results of principle experiment. (**a**) Original images (upper) and binarized images (lower) of the 12 μm single polystyrene sphere in the microchannel taken with an irradiation time of 4994 (left) and 2500 μs (right). (i) Images of stationary (stopping) particle. (ii) Images of moving particles. Elongation of the particle shapes was different depending on the difference of irradiation time. (iii) Restored images after image processing based on the calculated velocity with this method. Bars, 15 μm (1 px = 0.704 μm). (**b**) The relationship between the preset rotation velocity of the beam chopper (particle) and the measured velocity based on the principle (Plotted data and bars are mean values and standard deviations: N = 51, 53, 52, and 54 samples for 483.7, 725.4, 967.0, and 1208.3 mm/s of setting velocities, respectively). The dashed line indicates the linear fitting result ($y=0.8982x+122.62,R^{2}=0.952$).

Figure 3a shows an example of a set of images of the single microparticle fixed in the channel. The upper two bright-field images are the micrographs of 590-nm with 4994 μs (left) and 530-nm with 2500 μs (left) (right) lights. These two images were acquired simultaneously as a single shot image in a CCD-camera. They were divided from the single-shot image of the particle with dichroic mirrors in the multi-view module. The lower two micrographs are their binarized images to measure their elongation automatically on a computer.

As shown in the images in Figure 3a (i), the acquired images of fixed particle shapes with different wavelengths and exposure time showed the same spherical shape of the single 12 μm polystyrene sphere. When we apply the 20× obj. lens with HXC13 CCD camera, the binarized data values of both wavelength images were the same and were 21 px in diameter for the *X*-axis (perpendicular to the flow direction) and 21 px in diameter for *Y*-axis (parallel to flow direction). The area of the particle was 360 px. As the size of one pixel was 0.704 μm, the diameter of the particles can be measured as 14.7 μm. The results confirmed no significant difference in image size and length caused by the difference in the wavelength of lights.

Although the beads were spherical during their fixed stationary position (stopping) (Figure 3a (i)), elongated particle images were acquired due to the movement of the particles during the light exposure time (Figure 3a (ii)). When the microbead moved to Y-direction (flow direction) with 1.4 mm/s mechanically by rotation of the beam chopper, the binarized images were changed to 19 (*X*-axis), 28 (*Y*-axis), and 472 px (area) for the left image (590 nm with 4994 μs), and 19 (*X*-axis), 23 (*Y*-axis), and 359 px (area) for the right image (530 nm with 2500 μs) (see Figure 3a (ii)). The difference of the length of particles caused by the different irradiation time of lights was used for estimating the velocity of particle movement and was $\left(28-23\right)/\left(4994-2500\right)\times 0.704=1.41\times 10^{-3}$ [m/s], which was consistent with the rotation velocity. It should be noted that the *X*-axis length of both particles was similarly shrunk by 2 px, which should not be changed in this experiment because this direction is perpendicular to the movement direction. It may be caused by the image blur of the total shape of the particle and might have changed the edge of binarized images of particles. In this experiment, we did not add any edge enhancement technology into the edge detection and applied same threshold value for moving particles as the stopped particles. When we applied more precise edge detection technology, more precise binarized data analysis can be adopted for this velocity measurement.

As shown in the images of Figure 3a (ii), the difference of elongation ratio depended on the difference of irradiation time of two-wavelength lights. In other words, the exposure time changes the shapes of the sample in images. However, using the acquired velocity information, we can restore the original shape of the samples by exploiting the idea explained in Figure 1b (iv). The principle-based reconstruction was shown in Figure 3a (iii). The pixels of the lower half in the flow direction of the raw image of the sample was lifted to upstream direction by 10 px in one by one manner for the left image and by 5 px for the right image, where the values 10 px and 5 px were acquired from the calculation of the shift of the lower edge position pixels during the 4994 μs and 2500 μs exposure time with a flow velocity of particle, 2 px/ms.

Figure 3b demonstrates the result of the accuracy of the velocity measurement of this principle. The velocity of movement of the microparticle on the rotating beam chopper was observed at 0, 483.7, 725.4, 967.0, and 1208.3 mm/s, and the irradiation time of lights was set at 2500 and 4994 μs for 530 and 590 nm lights, respectively. Exploiting the velocity measurement procedure described above, we plotted the relationship between the set rotation velocity of the beam chopper and the measured velocity of the 12 μm polystyrene spheres. The results exhibited a linear correlation between the set velocities and measured velocities. Therefore, the result authenticated that the particle velocity can be measured with the single-shot of two-wavelength images using this method.

### 4.2. Flow Velocity Distribution in Microfluidic Pathway

Next, we examined the ability of flow velocity measurement of microparticles within the microchannel with shingle-shot image acquisition. In this experiment, 75 μL of the sample was applied to the sample inlet of the microchip in the system. When the sample was microbeads, pure water was used as the buffer solution and sheath buffer, and when the sample was cells, the cell suspension was used as a sheath buffer. Air pressure was applied to both sample and sheath buffer inlets equally and simultaneously using a syringe pump to control the flow speed of samples (Figure 2e). The microchip was illuminated by a halogen lamp that emits light continuously and an LED light with a 2.5 ms flushing every 5 ms intervals synchronized with the shutter opening timing of the CCD camera.

**Figure 4:**
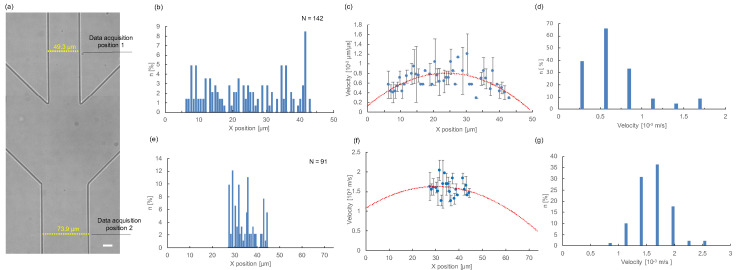
Measurement results of the velocity of microbeads flowing in the microchannel. (**a**) Microchannel and two data acquisition positions. The flow path widths for the upper stream (data acquisition positions 1) and the lower stream (data acquisition position 2) were 49.3 and 73.9 μm, respectively. The depth was 19.9 μm. Bar, 15 μm (1 px = 0.704 μm). (**b**) Positional distribution of 142 passing particles at the upper stream (data acquisition position 1). We counted the center coordinates of the particles. (**c**) Relationship between X position and measured velocity of 142 particles at the upper stream (data acquisition position 1). Plots show the average velocity and standard deviation of particles at each X position. The dashed line indicates the quadratic approximation curve ($y=-0.0012x^{2}+0.0567x+0.1255,R^{2}=0.350$). (**d**) Velocity distribution of whole 142 particles at the upper stream (data acquisition position 1). (**e**) Positional distribution of 91 passing particles at the lower stream (data acquisition position 2). (**f**) Relationship between X position and measured velocity of 91 particles at the lower stream (data acquisition position 2). Plots show the average velocity and standard deviation of particles at each X position. The dashed line indicates the quadratic approximation curve ($y=-0.0006x^{2}+0.0364x+1.0808,R^{2}=0.037$). (**g**) Velocity distribution of whole 91 particles at the lower stream (data acquisition position 2).

Figure 4 shows the measurement results of the velocity of 12 μm microbeads flowing in the microchannel. Figure 4a is an image of the PDMS microchannel, indicating the two data acquisition positions: one (data acquisition position 1) was the upper stream of sample inlet pathway before the junction of hydrodynamic focusing, and the other (data acquisition position 2) was the lower stream after hydrodynamic focusing where the sheath side buffers were expected to concentrate the samples into the center of the microchannel. Particles that have passed through these positions were observed and stored as captured images into the computer.

Figure 4b shows the spatial distribution of passed 142 particles at the upper stream (data acquisition position 1). The distribution demonstrates that the flow of the particles was dispersed all over the microfluidic pathway width. Figure 4c shows the spatial distribution of the measured velocity of 142 particles in the microfluidic pathway (X position) at the upper stream (data acquisition position 1). The mean values and standard deviations of particles were plotted in the graph, and the dashed line was the curve fitting result. As described in the dashed line, the obtained results were consistent with the expected laminar flow in the microchannel. Figure 4d is the velocity distribution of 142 particles at the upper stream (data acquisition position 1). As the histogram indicates, a wide variety of flow velocity exists in the microchannel.

Figure 4e shows the spatial distribution of passing 91 particles at the lower stream (data acquisition position 2). It was confirmed that the particles were centered by the hydrodynamic focusing of two side sheath buffer flows. Figure 4f shows the spatial distribution of the measured velocity of 91 particles in the microfluidic pathway (X position) at the lower stream (data acquisition position 2). The mean values and standard deviations of particles were plotted in the graph, and the dashed line was the curve fitting result. Figure 4g shows the velocity distribution of 91 particles at the lower stream (data acquisition position 2). Although the spatial distribution of the particles was focused on the center of microchannel by the hydrodynamic focusing, the flow velocity distribution still remained. This result suggests that the spatial focusing was not sufficient to reduce the distribution of flow velocities of particles.

From the above results, we can indicate our system was able to measure the velocity of particles in the microchannel with only one shot. In addition, we found that the maximum flow velocity of microparticles differs depending on the X position in the channel, and even though the X position was the same, the flow velocity still varied even after the hydrodynamic focusing. Based on these results, we can suggest that it is necessary for accurate flow velocity measurement of each particle for precise cell sorting downstream because we need to estimate the correct sorting time to shift a particular target particle at the sorting point with the accurate flow velocity.

### 4.3. Simultaneous Flow Velocity Measurement of Particles in Microfluidic Pathway

Another advantage of this flow velocity measurement method is the simultaneous measurement of the plurality of particles with a single shot of image acquisition. We examined the ability of this simultaneous velocity measurement of particles.

**Figure 5:**
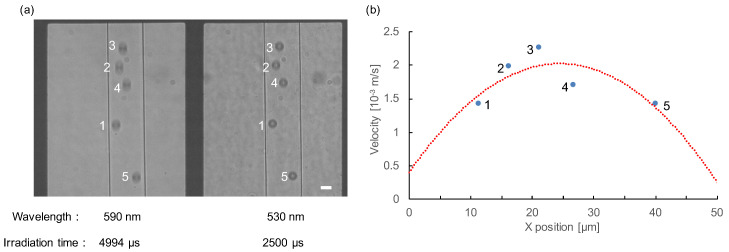
Velocity measurement of simultaneously flowing five particles. (**a**) The image was taken when multiple particles flowed simultaneously in the microchannel. Two-wavelength images of five particles were observed. The elongation was different depending on the difference of their flow velocities. Bar, 15 μm (1 px = 0.704 μm). (**b**) Measurement results of the five observed particle velocities. The velocity was dependent on the location of microchannel. The red dashed line is the quadratic approximation curve of laminar flow ($y=-0.0027x^{2}+0.133x+0.3987,R^{2}=0.610$).

Figure 5 shows an example of the velocity measurement of the plurality of particles in a shot of image acquisition flowing in the microchannel. Figure 5a shows a photograph of five particles flowing in the microchannel simultaneously within the image (left, 4994 μs exposure of 590 nm image, and right, 2500 μs of 530 nm image). Figure 5b is the relationship between the width (X) position and the velocity of observed five particles. From this result, the velocity distribution of microparticles were followed by the parabolic laminar flow distribution. As described above, we showed that our system can measure the velocities of all particles within a single shot of an image.

### 4.4. Precise Size Measurement with Flow Velocity Correction of Particles in Microfluidic Flow

Imaging flow cytometry uses the images of particles to identify target samples instead of their indirect information, such as the intensity of diffraction or fluorescence. Hence, the precise measurement of the image-based index, including actual size or a particular shape, is essential. However, because of the movement of samples during the exposure time, the acquired images of samples were elongated to the flow direction as far as the exposure time exists. To overcome this problem, our single-shot velocity measurement method can also give us another advantage for improving the accuracy of sample sizes. When we can acquire the precise flow velocity of each particle, we can reconstruct the shape information of the particle based on this displacement information—as explained in Figure 1b (iv).

**Figure 6:**
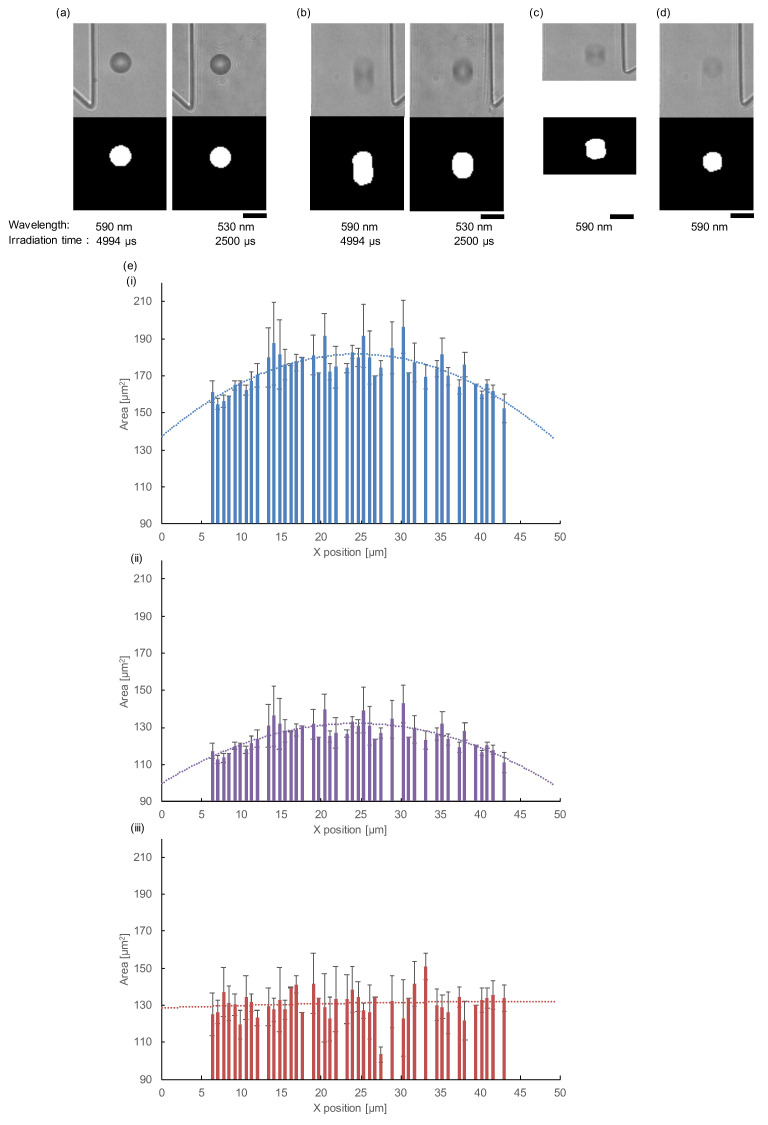
Result of exact shape restoration process considering the particle’s velocity. (**a**) Images of a stationary (stopping) particle (upper) and its binarized images (lower). (**b**) Images of flowing particles. Bright-field images (upper) and binarized images (lower). (**c**) Images obtained from conventional restoration method, which compresses the elongated particle image only considering the flow velocity. The elongated shape was equally shortened in the flow direction. (**d**) Images obtained from the restoration processing considering velocity and exposure time, as explained in Figure 1b (iv). The acquired shape of the particle was recovered to the round shape and was similar to the stationary (stopping) particle. Bars, 15 μm (1 px = 0.704 μm). (**e**) Relationship between the position in the microchannel (*X*-axis) position and the area of 142 microbeads shown in Figure 4b–d. (i) The raw image data of particle area distribution. The areas of particles were correlated to the flow velocities of those particles. Hence, the raw data of particle area distribution was well fit to the parabolic curvature (dashed line: $y=-0.0744x^{2}+3.6406x+137.26,R^{2}=0.615$). (ii) The corrected particle area distribution only considering the flow velocity. Dashed line was the averaged area of particles acquired and still shows a quadratic curve ($y=-0.0541x^{2}+2.6477x+99.826,R^{2}=0.615$). (iii) The corrected particle area distribution considering velocity and exposure time. The dashed line was the mean value of the corrected area ($-0.0015x^{2}+0.1404x+128.64,R^{2}=0.010$) and was almost flat regardless of the place in the microchannel.

Figure 6 shows the result of the actual shape restoration process of the same 142 samples of Figure 4b–d. Figure 6a shows the images of a stationary (stopping) particle in 590 and 530 nm wavelengths (upper) and their binarized images (lower). These binarized images of the 12 μm microbead in 530 and 590 nm wavelengths were round-shaped. Figure 6b shows images of a moving (elongated) particle and its binarized pictures in 530 and 590 nm wavelengths. The difference in elongation lengths was caused by the difference in the exposure time, 4994 and 2500 μs. From the difference in particle’s elongation length and the difference in irradiation time of these two light sources, the velocity of this particle was determined as 1.69 $\times 10^{-3}$ m/s. Figure 6c shows images obtained by the conventional restoration method, which just compressed the raw image of the flow direction (*Y*-axis) to recover the amount of elongation that occurred during the exposure time. The irradiation time of the light having a wavelength of 590 nm is 4994 μs. By using the irradiation time of the light and the calculated velocity (1.69 $\times 10^{-3}$ m/s), the particle’s elongation was identified to be 8.44 μm. Since the length of the elongated particles was 14.8 μm, As shown in Figure 6c, the elongated shape of the particle was recovered to 12 μm in the flow direction, which was obtained with the magnification of the elongated particle image at 8.44/14.8 in the flow direction. However, the shape of the particle was different from the spherical microbead. Hence, we can conclude that the conventional simple image reduction method fails to restore the original shape of the particles.

Figure 6d is the image obtained from the restoration method we proposed in Figure 1b (iv), in which the lower parts of the elongated particle were lifted by the amount of elongation determined by multiplying the calculated velocity and irradiation time. As shown in the images, the original shape and size of the circular particle have been restored appropriately. Figure 6e shows the relationship between the X position and the averaged area of 142 samples of Figure 4b–d. In general, as shown in Figure 6e (i), even though all the particle sizes were 12 μm, the acquired raw area data of those samples were larger when the velocity of the particles was faster. When we applied the conventional method to all elongated particle images, the distribution of the difference in their areas was shown depending on the X position, and it can be seen that this method cannot restore the correct shape (Figure 6e (ii)). On the other hand, when we applied our restoration method to the particles, the distribution of particle areas became flat (Figure 6e (ii)). These results indicate that the importance of the correct restoration method to acquire accurate area information and also the importance of the acquisition of precise velocity information of each particle for its restoration.

### 4.5. Ability and Potential Limitation of This Method for Imaging Flow Cytometry Measurement

In this paper, we proposed the simple single-shot image-based velocity measurement method and its application for size correction. This method exploited the exposure time for image acquisition for velocity analysis. Usually, the exposure time is necessary to the image acquisition and the origin of quality reduction of images, namely image blur. However, focusing on the bright side of these characteristics enables us to acquire the precise measurement of velocity and size of samples as described above in this paper.

In principle, this method is simple and can be applied for all the measurements. However, the resolution and preciseness are limited to the ability and resolution of hardware. From this viewpoint, the ability of this method is strongly reliant on the progress of the technologies.

**Figure 7:**
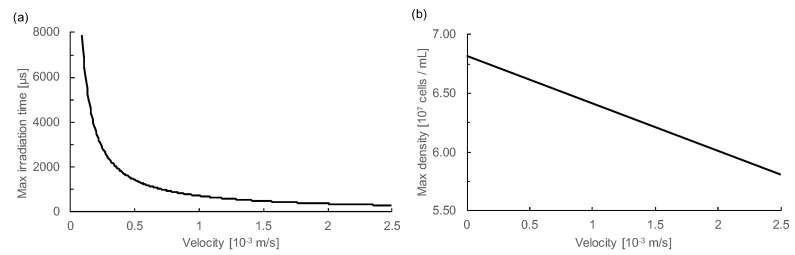
The potential limitation of this method. (**a**) Relationship between particle velocity and maximum irradiation time. The maximum irradiation time is the time required for a particle to move 1 px (1 px = 0.704 μm). If the irradiation time is shorter than that time, it is possible to obtain an image of the particles without stretching. (**b**) Relationship between particle velocity and the maximum density of 12 μm beads. This refers to a density that does not allow multiple particles to appear in the 80 × 80 px image that is cropped. The irradiation time was set to 4994 μm, and the elongation of the particles due to this was taken into account.

First, we focused on the limitations of camera resolution. If the moving distance of the particle is less than the minimum unit of resolution (1 px) during the light irradiation time, the image of the particle is obtained without stretching. Figure 7a shows the relationship between particle velocity and maximum irradiation time. Capturing the particles without elongation is possible by setting the irradiation time below the time of the curve shown in the figure. On the other hand, since the velocity is calculated using the difference in the particle’s *Y*-axis lengths, the difference between the irradiation times of the two wavelengths needs to be longer than the maximum irradiation time.

Next, we considered the influence on sorting accuracy. If multiple particles appear in the image clipped to 80 × 80 px, accurate particle recognition cannot be performed. Therefore, it is necessary to adjust the density and velocity of the sample particles. Figure 7b shows the relationship between the speed and maximum density of 12 μm beads at the data acquisition position 2. The minimum distance that prevents neighboring particles from being simultaneously captured was calculated and converted to density. For this purpose, the elongation of the particle, determined from the velocity and the irradiation time, was taken into account. Shutter time was set to 4994 μs. A depth of 19.9 μm, a mean X position of the flowing particles of 34.4 μm, and a standard deviation of 5.31 μm at data acquisition position 2 (Figure 4f) were used in the calculation. In order to perform an accurate measurement, it is necessary to measure the sample at a density lower than the max density indicated by the straight line in the figure.

### 4.6. Accurate Shape Reconstruction of Flowing HeLa Cells

**Figure 8:**
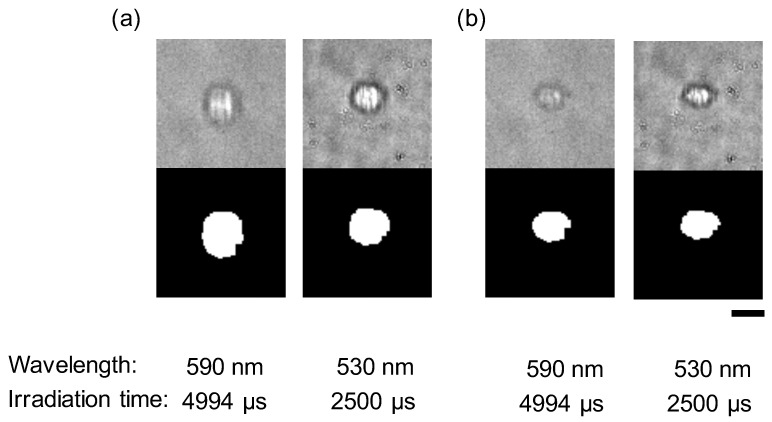
Results of a restoration of a flowing HeLa cell. (**a**) Images of a flowing HeLa cell (upper) and its binarized images (lower) at data acquisition position 2. Bar, 15 μm (1 px = 0.704 μm). (**b**) Images obtained from the restoration processing considering velocity and exposure time.

Finally, we applied this method for practical living samples by using HeLa cells. Figure 8 shows images of a flowing HeLa cell at the data acquisition position two (see Figure 4a) and its restored images in 590 and 530 nm wavelengths. The acquired cell’s velocity was 1.41 $\times 10^{-3}$ m/s. The area of the cell in the captured image (590 nm) was 293.5 μm${}^{2}$, whereas its size was corrected to 170.6 μm${}^{2}$ after restoration. This example shows the ability of this method for the living cells.

Recently the reconstruction technology of 3-D images of cells and their clusters by their rotations in the microfluidic device was developed [18]. Our precise cell flow velocity can give precise displacement information adding to the rotation images of particles to reconstruct those 3-D images even during flowing.

## 5. Conclusions

We examined our simple single-shot measurement of the flow velocity of samples and its application for sample size restoration in standard 12 μm polystyrene spheres and HeLa cells. This method exploited the positive side of exposure time of images and can obtain all the sample velocities and corrected area size information simultaneously with a single image acquisition shot. Although the resolution and preciseness of acquired velocity and area information were limited by the ability and resolution of existing hardware, in principle, this method can be applied for all the imaging flow cytometry, and also the ability and resolution can be improved according to the improvement of hardware. Especially the advantage of this method, precise measurement of the flow velocity of single particles within a flow, can be applied for correction and reconstruction of images such as high-throughput reconstruction of 3-D images of flowing cell clusters by their rotations as the next step of this application. We think this method can contribute to the improvement of general imaging flow cytometry for more precise target recognition and also for correct target collection timing decisions especially for the diagnostic screening including circulation tumor cells.

## Abbreviations

The following abbreviations are used in this manuscript:

CAD	computer-aided design
CTC	circulating tumor cell
DMEM	Dulbecco’s modified Eagle medium
FBS	fetal bovine serum
LED	light emitting diode
PDMS	polydimethylsiloxane
px	pixel

## References

[1] Chou H.P., Spence C., Scherer A., Quake S. (1999). A microfabricated device for sizing and sorting DNA molecules. Proc. Natl. Acad. Sci. USA.

[2] Cheung K., Gawad S., Renaud P. (2005). Impedance spectroscopy flow cytometry: On-chip label-free cell differentiation. Cytom. Part A.

[3] Huh D., Gu W., Kamotani Y., Grotberg J.B., Takayama S. (2005). Microfluidics for flow cytometric analysis of cells and particles. Physiol. Meas..

[4] Cheung K.C., Berardino M.D., Schade-Kampmann G., Hebeisen M., Pierzchalski A., Bocsi J., Mittag A., Tárnok A. (2010). Microfluidic impedance-based flow cytometry. Cytom. Part A.

[5] Bow H., Pivkin I.V., Diez-Silva M., Goldfless S.J., Dao M., Niles J.C., Suresh S., Han J. (2011). A microfabricated deformability-based flow cytometer with application to malaria. Lab Chip.

[6] Karabacak N.M., Spuhler P.S., Fachin F., Lim E.J., Pai V., Ozkumur E., Martel J.M., Kojic N., Smith K., Chen P.I. (2014). Microfluidic, marker-free isolation of circulating tumor cells from blood samples. Nat. Proc..

[7] Stott S.L., Hsu C.H., Tsukrov D.I., Yu M., Miyamoto D.T., Waltman B.A., Rothenberg S.M., Shah A.M., Smas M.E., Korir G.K. (2010). Isolation of circulating tumor cells using a microvortex-generating herringbone-chip. Proc. Natl. Acad. Sci. USA.

[8] Hosokawa M., Yoshikawa T., Negishi R., Yoshino T., Koh Y., Kenmotsu H., Naito T., Takahashi T., Yamamoto N., Kikuhara Y. (2013). Microcavity array system for size-based enrichment of circulating tumor cells from the blood of patients with small-cell lung cancer. Anal. Chem..

[9] Yu B.Y., Elbuken C., Shen C., Huissoon J.P., Ren C.L. (2018). An integrated microfluidic device for the sorting of yeast cells using image processing. Sci. Rep..

[10] Utharala R., Tseng Q., Furlong E.E., Merten C.A. (2018). A Versatile, Low-Cost, Multiway Microfluidic Sorter for Droplets, Cells, and Embryos. Anal. Chem..

[11] Sesen M., Whyte G. (2020). Image-Based Single Cell Sorting Automation in Droplet Microfluidics. Sci. Rep..

[12] Nitta N., Sugimura T., Isozaki A., Mikami H., Hiraki K., Sakuma S., Iino T., Arai F., Endo T., Fujiwaki Y. (2018). Intelligent Image-Activated Cell Sorting. Cell.

[13] Ota S., Horisaki R., Kawamura Y., Ugawa M., Sato I., Hashimoto K., Kamesawa R., Setoyama K., Yamaguchi S., Fujiu K. (2018). Ghost cytometry. Science.

[14] Andree K.C., van Dalum G., Terstappen L.W. (2016). Challenges in circulating tumor cell detection by the CellSearch system. Mol. Oncol..

[15] Hao S.J., Wan Y., Xia Y.Q., Zou X., Zheng S.Y. (2018). Size-based separation methods of circulating tumor cells. Adv. Drug Deliv. Rev..

[16] Huang X., Tang J., Hu L., Bian R., Liu M., Cao W., Zhang H. (2019). Arrayed microfluidic chip for detection of circulating tumor cells and evaluation of drug potency. Anal. Biochem..

[17] Shibuta M., Tamura M., Kanie K., Yanagisawa M., Matsui H., Satoh T., Takagi T., Kanamori T., Sugiura S., Kato R. (2018). Imaging cell picker: A morphology-based automated cell separation system on a photodegradable hydrogel culture platform. J. Biosci. Bioeng..

[18] Puttaswamy S.V., Bhalla N., Kelsey C., Lubarsky G., Lee C., McLaughlin J. (2020). Independent and grouped 3D cell rotation in a microfluidic device for bioimaging applications. Biosens. Bioelectron..

[19] Takahashi K., Hattori A., Suzuki I., Ichiki T., Yasuda K. (2004). Non-destructive on-chip cell sorting system with real-time microscopic image processing. J. Nanobiotechnol..

[20] Hayashi M., Hattori A., Kim H., Terazono H., Kaneko T., Yasuda K. (2011). Fully automated on-chip imaging flow cytometry system with disposable contamination-free plastic re-cultivation chip. Int. J. Mol. Sci..

[21] Yasuda K., Hattori A., Kim H., Terazono H., Hayashi M., Takei H., Kaneko T., Nomura F. (2013). Non-destructive on-chip imaging flow cell-sorting system for on-chip cellomics. Microfluid. Nanofluid..

[22] Girault M., Kim H., Arakawa H., Matsuura K., Odaka M., Hattori A., Terazono H., Yasuda K. (2017). An on-chip imaging droplet-sorting system: A real-time shape recognition method to screen target cells in droplets with single cell resolution. Sci. Rep..

[23] Kim H., Terazono H., Nakamura Y., Sakai K., Hattori A., Odaka M., Girault M., Arao T., Nishio K., Miyagi Y. (2014). Development of on-chip multi-imaging flow cytometry for identification of imaging biomarkers of clustered circulating tumor cells. PLoS ONE.

[24] Hattori A., Kim H., Terazono H., Odaka M., Girault M., Matsuura K., Yasuda K. (2014). Identification of cells using morphological information of bright field/fluorescent multi-imaging flow cytometer images. Jpn. J. Appl. Phys..

[25] Odaka M., Kim H., Nakamura Y., Hattori A., Matsuura K., Iwamura M., Miyagi Y., Yasuda K. (2019). Size distribution analysis with on-chip multi-imaging cell sorter for unlabeled identification of circulating tumor cells in blood. Micromachines.

